# Singing in the Rain Forest: How a Tropical Bird Song Transfers Information

**DOI:** 10.1371/journal.pone.0001580

**Published:** 2008-02-13

**Authors:** Nicolas Mathevon, Thierry Aubin, Jacques Vielliard, Maria-Luisa da Silva, Frédéric Sebe, Danilo Boscolo

**Affiliations:** 1 Equipe ‘Communications Acoustiques’ Neurobiologie de l'Apprentissage, de la Mémoire et de la Communication (NAMC) Centre National de la Recherche Scientifique (CNRS) UMR8620, Université Paris XI, Orsay, France; 2 Laboratoire ‘Ecologie & Neuro-Ethologie Sensorielles’ EA 3988, Université Jean Monnet, Saint-Etienne, France; 3 Laboratório de Bioacústica, Instituto de Biologia, Universidade Estadual de Campinas, Campinas, São Paulo, Brazil; 4 Centro de Ciências Biológicas, Universidade Federal do Pará, Campus Universitário do Guamá, Belém, Pará, Brazil; Laboratório de Ecologia da Paisagem e Conservação, Instituto de Biologia, Universidade de São Paulo, São Paulo, Brazil; University of Sussex, United Kingdom

## Abstract

How information transmission processes between individuals are shaped by natural selection is a key question for the understanding of the evolution of acoustic communication systems. Environmental acoustics predict that signal structure will differ depending on general features of the habitat. Social features, like individual spacing and mating behavior, may also be important for the design of communication. Here we present the first experimental study investigating how a tropical rainforest bird, the white-browed warbler *Basileuterus leucoblepharus*, extracts various information from a received song: species-specific identity, individual identity and location of the sender. Species-specific information is encoded in a resistant acoustic feature and is thus a public signal helping males to reach a wide audience. Conversely, individual identity is supported by song features susceptible to propagation: this private signal is reserved for neighbors. Finally, the receivers can locate the singers by using propagation-induced song modifications. Thus, this communication system is well matched to the acoustic constraints of the rain forest and to the ecological requirements of the species. Our results emphasize that, in a constraining acoustic environment, the efficiency of a sound communication system results from a coding/decoding process particularly well tuned to the acoustic properties of this environment.

## Introduction

Acoustic communication systems, such as birdsongs or human language, are likely to be shaped by natural selection [1]. Environmental and social constraints represent potential forces influencing how information is encoded in acoustic signals by the sender as well as how perceived sounds are processed by the receiver [2]–[6]. Although this is a key question for the understanding of communication processes, the identification of these constraints is problematic because, 1) most natural systems fluctuate unpredictably and, 2) presently observed biological phenomena are likely to be the result of a complicated and chaotic evolutionary history [7]. In tropical rain forests, evolutionary constraints upon sound communication due to dense vegetation have likely been stable over the past millennia up to present [8], [9]. This represents a unique opportunity to link present biological processes with environmental constraints that have remained the same for ages. This paper describes how the information transfer supported by the simple and stereotyped song of a typical bird of the Brazilian Atlantic rainforest, the white-browed warbler, *Basileuterus leucoblepharus* (Aves: Passeriformes: Fringillidae: Parulini), is well matched to the acoustic propagation constraints of the rainforest and to the ecological requirements of this species.

**Figure 1:**
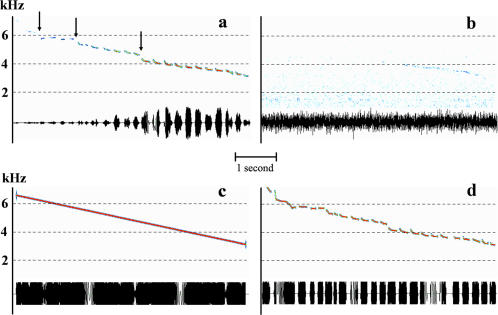
Territorial song of the white-browed warbler. a, Control song recorded at 1 m. Arrows indicate “gaps” in frequency. b, Song recorded after a 100 m propagation through the tropical forest. c, Synthetic pure tone mimicking the natural slope of frequency modulation. d, song without amplitude modulation.

The white-browed warbler is a common Brazilian bird living in the undergrowth (0–6 m high) of the Atlantic forest where visual communication is strongly limited by obstacles [10]. Thus sound communication is a key component for social interactions in this species. The acoustic repertoire of the male consists mainly of two signals: a high-pitched contact call and a territorial song which appears very stereotyped among individuals (succession of similar notes - duration : 5–6 seconds - slowly decreasing in frequency from 7 kHz to 3 kHz, Fig. 1a, audio S1). Males are monogamous and actively defend territories (diameter: about 75–100 meters) against conspecifics all year long [11]. As in many other songbirds [12], the song is used for both female attraction and territorial interactions. Since vocalizations are high-pitched, they are unlikely to propagate far through the dense vegetation of the rainforest [12]–[15]. In spite of this environmental constraint, the song must establish a reliable communication between individuals. Two types of potential information encoded in the song are of primarily biological importance for a receiving bird: species-specific information to recognize a potential rival or partner and individual information to allow a territory owner to identify its established neighbors versus stranger birds [12], [16]. In addition, it is also advantageous to identify the location of the sender to assess the competitor's threat since a remote rival is less dangerous than a near one [17].

In the current experimental study, our prediction is that the simple song of the white-browed warbler is able to efficiently support various information - species identity, individual identity and location of the sender - in spite of the highly constraining environment of the tropical rain forest.

## Results

### Propagation-induced song modifications

We first examined the environmental constraints impinging on the white-browed warbler's acoustic communication by analyzing the modifications affecting the song during its transmission through the forest using propagation experiments. The striking result is that the first half of the signal corresponding to the higher frequencies is embedded in the background noise after 100 m of propagation (Fig. 1b, audio S2)[18]. In addition to this propagation-induced shortening of the emitted song, amplitude and frequency modulations are also modified (correlations between control and propagated signals for amplitude envelope: at 25 m, r = 0.84; at 50 m, r = 0.56; at 100 m, r = 0.36; spectrographic cross-correlation between control and propagated signals: at 25 m, r = 0.93; at 50 m, r = 0.91; at 100 m, r = 0.68; all *p*<0.01, *n* = 6).

### Species-specific information

**Figure 2:**
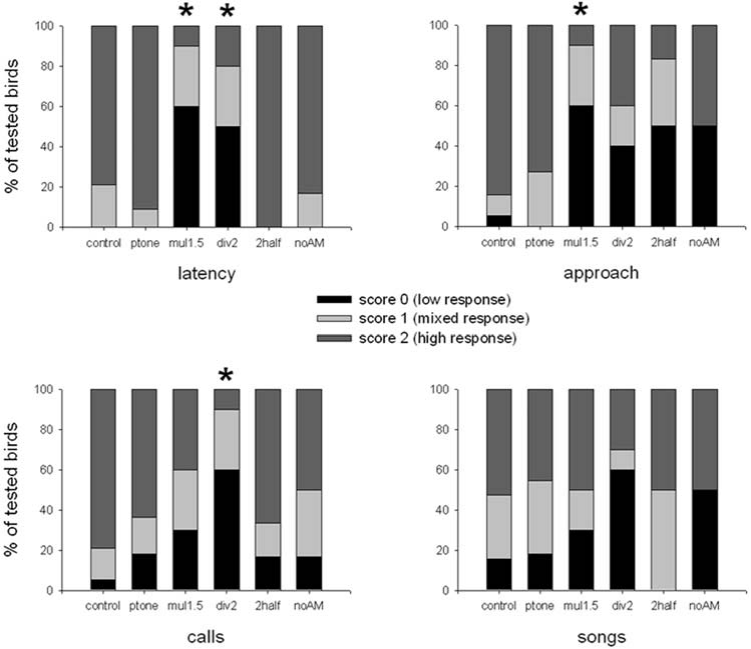
Behavioral responses to experimental signals. (control = control song, *n* = 19 birds; ptone = synthetic continuous pure tone, *n* = 11; mul1.5 = slope multiplied by 1.5, *n* = 10; div2 = slope divided by 2, *n* = 10; 2half = second half of the song, *n* = 6; noAM = without AM song, *n* = 6). Results of Krukal-Wallis tests: latency, *H (5, n = 62)* = 34.38, *P* = 0.000; approach, *H (5, n = 62)* = 22.24, *P* = 0.000; calls, *H (5, n = 62)* = 15.94, *P* = 0.007; songs, *H (5, n = 62)* = 4.55, *P* = 0.473. *: significant difference with control (post-hoc multiple comparison tests; see text for details).

**Figure 3:**
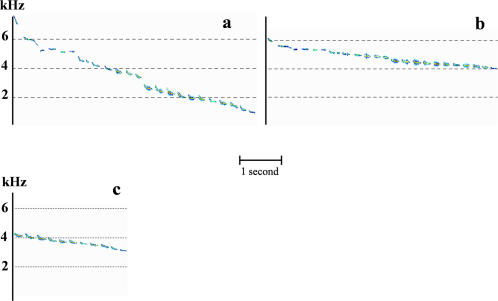
Experimental songs with modified FM slope or modified duration. a, FM slope multiplied by 1.5. b, FM slope divided by 2. c, second half of the song.

To understand how the territorial song supports species-specific recognition, we performed playback experiments with acoustic lures. Preliminary tests (*n* = 4) showed that the emission of a control song - recorded from a remote individual - from inside a male's territory always elicits a strong reaction from the owner, consisting of songs in reply and approach toward the sound source. To identify which acoustic parameter(s) support(s) the species-specific message, we synthesized experimental signals with acoustic features of the original song selectively modified and performed playback tests. We found that species-specific recognition relies on the slope of the frequency modulation of the carrier frequency. A synthetic continuous pure tone mimicking the natural decreasing frequency slope of the carrier of the natural song (Fig. 1c, audio S3) induces a strong territorial behavior from challenged males (Fig. 2; post-hoc multiple comparisons revealed no significant difference between the control song and a synthetic pure tone: latency of response, *Z* = 0.446, *p* = 1.000, *n* = 11; approach to the speaker, *Z* = 0.266, *p* = 1.000, *n* = 11; calls emission rate, *Z* = 0.802, *p* = 1.000, *n* = 11; songs emission rate, *Z* = 0.294, *p* = 1.000, *n* = 11). Conversely, when all the acoustic features of the song are kept at their natural values except the frequency slope, the behavioral response decreases. Males showed a weak tolerance towards modifications of this parameter since modifications of the slope (multiplied by 1.5: audio S4, or divided by 2: audio S5, Fig. 3a,b) impair the species-specific recognition process (Fig. 2; control song compared with experimental song with FM slope multiplied by 1.5, post-hoc multiple comparisons: latency of response, *Z* = 3.47, *p*<0.01, *n* = 10; approach to the speaker, *Z* = 3,49, *p*<0.01, *n* = 10; calls emission rate, *Z* = 1.82, *p* = 1.000, *n* = 10; songs emission rate, *Z* = 0.417, *p* = 1.000, *n* = 10; control song compared with experimental song with FM slope divided by 2, post-hoc multiple comparisons: latency of response, *Z* = 2.95, *p*<0.05, *n* = 10; approach to the speaker, *Z* = 2.13, *p* = 0.503, *n* = 10; calls emission rate, *Z* = 3.43, *p*<0.01, *n* = 10; songs emission rate, *Z* = 1.76, *p* = 1.000, *n* = 10). We also found that the whole song is not necessary. If only the second half of the song was played-back (audio S6, Fig. 3c), males showed a territorial response (Fig. 2; post-hoc multiple comparisons revealed no significant difference between control compared with experimental song: latency of response, *Z* = 0.64, *p* = 1.000, *n* = 6; approach to the speaker, *Z* = 2.57, *p* = 0.154, *n* = 6; calls emission rate, *Z* = 0.54, *p* = 1.000, *n* = 6; songs emission rate, *Z* = 0.24, *p* = 1.000, *n* = 6). Thus, white-browed warbler males are highly tolerant towards song simplification in species-specific recognition and seem to disregard parameters that are substantially altered during transmission: the duration of notes and silences, the fine intra-note frequency modulation and the amplitude modulation. Moreover, they do not need to hear the first half of the song, which is likely to disappear in the background noise. We conclude that the relevant parameter is the average slope of the slow frequency modulation of the carrier frequency, a propagation-resistant parameter, enabling species identity information to be transmitted at long range.

### Individual identity information

**Figure 4:**
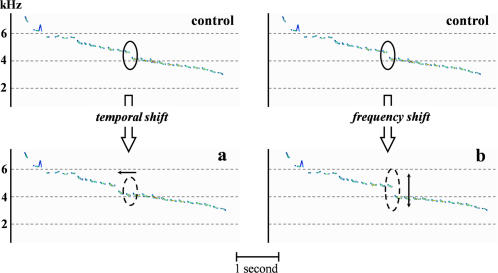
Experimental songs with the position of the main frequency “gap” modified. a, in the time domain. a, in the frequency domain.

Within the first half of the song, the frequency slope is disrupted by gaps in frequency between notes (arrows in Fig. 1a). The temporal position as well as the frequency interval of these gaps are remarkably similar within the songs of an individual while the number of gaps and their characteristics differ between individuals [18]. We deciphered the individual coding-decoding process by playback experiments. Males respond less aggressively to a neighbors' song than to an unfamiliar song (sign test, *p*<0.01, *n* = 9). As a result of playback experiments, a modification of the position of the main gap impaired the recognition of a neighbor's song (control neighbor's song compared with experimental neighbor's song with gap shifted in time, sign test, *p*<0.05, *n* = 6; control neighbor's song compared with experimental neighbor's song with gap shifted in frequency, sign test, *p*<0.05, *n* = 5; audios S7 and S8; Fig. 4). After long range transmission, the first half of the signal, and consequently the individual vocal signature, disappears into the background noise. Individuality is thus likely to be a privatized information with an “active space” restricted to the immediate surroundings of the emitting bird.

### Localization of the singer

#### Ranging

We tested if male white-browed warblers were able to estimate the location of a singing rival by relying on the degradation of the signal acoustic structure, an ability named « ranging » [17]. We thus assessed the response to a bird virtually positioned within or outside the border of the focal bird's territory using a two-loudspeaker experiment. We attracted the tested birds (n = 18) to the vicinity of a first loudspeaker positioned in the center of the territory by the emission of a control song. Once the tested bird remained positioned at less than 5 meters from the first loudspeaker, we played back an experimental signal (either the non-propagated control song or a 50m-propagated one) from a second loudspeaker situated 50 m farther (the average diameter of a territory is about 75–100 meters). The birds typically reacted by flying in the direction of the second loudspeaker. However, birds were more reluctant to leave their first position and flew a shorter distances in response to the 50m-propagated signal than to the non-propagated song (Fisher's exact tests: latency of departure from the first loudspeaker position, *p*<0.01, *n* = 18; approaching distance to the second speaker, *p*<0.05, *n* = 18). Thus, listening males range the emitter using sound degradation cues and modify their behavior accordingly – as a stimulus corresponding to a remote rival elicits less displacement than a near one. The particular structure of the song, a succession of short notes decreasing in frequency, may allow the receiver to assess the distance with a great precision. Due to the relative homogeneity of the tropical forest vegetation structure, the number of notes remaining after propagation must be directly correlated to transmission distance.

#### Azimuth assessment

In addition to ranging, birds must also have some ability to estimate the direction of the sound source in order to localize it. Previous research suggests amplitude modulation (AM) may be important for this azimuth assessment [19]. As a pilot experiment, we challenged birds in the center of their territories (n = 6 birds) with a song without AM (audio S9) to check that this signal retained its biological significance (i.e., species-specific territorial signal): males showed strong territorial responses with as many calls and songs compared with responses to control song (Fig. 2; post-hoc multiple comparisons: latency of response, *Z* = 0.13, *p* = 1.000, *n* = 6; approach to the speaker, *Z* = 1.68, *p* = 1.000, *n* = 6; calls emission rate, *Z* = 1.01, *p* = 1.000, *n* = 6; songs emission rate, *Z* = 0.74, *p* = 1.000, *n* = 6). In a playback experiment using the two-loudspeaker design (experimental signals: control song or without AM-song), we found that males responding to without natural AM-song did not delay their take off to the second loudspeaker in comparison with control. Interestingly, however, they made numerous flight direction errors not observed in response to control songs (Fisher's exact tests: latency of departure, *p* = 0.471, *n* = 18; approach to the second speaker, *p*<0.05, *n* = 18; choice of correct direction, *p*<0.05, *n* = 18), indicating that they were not able to accurately localize the second loudspeaker. We conclude that the precise acoustic localization of a bird is based at least on two factors: degradation cues which are « encoded » during transmission by a signal-environment interaction allowing ranging, and amplitude modulation characteristics of the emitted signal allowing azimuth assessment.

## Discussion

Natural selection is thought to play a powerful role in shaping animal acoustic communication. Among other factors, the physical environment is important in selecting for structural characteristics of sounds and the social environment is important in selecting for various form of coding complexity [20]–[25]. Focusing on sound transmission and signal detection, two main categories of signals can be distinguished: those transmitted at long range and those transmitted at short range [14]. In a parallel manner, studies focusing on communication networks categorize information as “public” when the sender advertises to a wide audience and as “private” when the active space of the signal is limited to a restricted number of receivers [26], [27]. During signal evolution, different pressures may arise in signal design depending on whether it is specialized to advertise or privatize information [26]. The white-browed warbler shows that these properties, long or short range transmission, and these functions, private or public information, are not mutually exclusive and can be supported by a unique song with a very simple structure. Thus, species-specific identity is a long-range message, encoded in a resistant acoustic feature –the slow descending modulation frequency. This « public » signaling may help males to reach a wide audience consisting of females to attract and erratic males to repel. Conversely, individual identity is a short-range message, supported by signal cues that are rapidly degraded during propagation –the frequency gaps of the higher pitched part. This « private » signaling is likely to be limited to neighbors of the sender. At the opposite of the transmission chain, the receivers have the possibility to assess the “status” (neighbors or strangers) and the localization of the singers, using both propagation-induced modifications and amplitude modulation cues.

Due to its dense vegetation, the tropical forest represents a challenging condition for the evolution of both productive and receptive components of acoustic communication systems. The sender's challenge is to control the « active space » of its signal either to maximize the audience or to target given individuals by coding the different categories of information in more or less ‘propagation-resistant’ sound parameters. From the receiver's point of view, the problem is to optimize the gained information in spite of potentially altered received sounds. A great tolerance to signal modifications as well as the use of propagation-induced modifications as location cues represent adapted decoding strategies. Our results emphasize that such strategies may have been developed in parallel to allow a communication system to be effective. Thus, the acoustic communication of the white-browed warbler is both finely tuned to environmental constraints and coherent with its way of life. With the song of this species, we show that, in a constraining acoustic environment, the information available to receivers for detecting, identifying and locating conspecifics results from an interaction between the structure of the produced signal and its transmission through the environment. This allows the establishment of efficient local communication networks in the particular habitat of a tropical forest.

While the biology of tropical birds differs in many ways from those of temperate areas, information on the communication behavior of tropical birds is limited [28]. Our study shows that the tropical rainforest ecosystem represents a unique opportunity to experimentally test theoretical models of acoustic communication. This underscores the critical importance of tropical areas for addressing fundamental biological questions and reinforces the urgency for protecting such major biodiversity hotspots as those of the Brazilian Atlantic forest [29].

## Materials and Methods

### Study site

The study was performed in the Atlantic forest of Southeastern Brazil (Morro Grande State Reserve), during November 2000 and 2001 (temperature: 16–22°C, relative humidity: 85–100%). The white-browed warbler is a small Parulidae, feeding on insects and living in the understory [11]. The species was chosen for its high abundance and regular singing activity. In the Morro Grande reserve, the white-browed warbler show a very high IPA- Index of Point Abundance and an estimated density is about 15–20 pairs per km^2^ [30, Vielliard, pers.obs.].

### Recording and playback procedures

Recordings of wild birds were performed using an ultra-directional Sennheiser MKH 816 microphone connected to a Sony TCD-D10 DAT recorder (recording distance: less than 6 meters from the bird; sampling frequency: 48 kHz; frequency response: flat within the range 20–20000 Hz) and subsequently digitised at a sampling frequency of 22 kHz. For both propagation and playback experiments, the emission chain was made of a Sony TCD-D7 DAT tape recorder connected to a 10W customized amplifier and an Audax loudspeaker (frequency response: 100–8600±2.5 dB; sounds emitted at 90 dB_SPL_ measured at 1 m from the loudspeaker with a Bruel & Kjaer sound level meter type 2235 equipped with a 1/2 inch 4126 microphone, slow setting, linear scale). For propagation experiments, the reception chain was made of an omnidirectional Beyer dynamic M69 TG microphone (frequency response 150–15000 Hz±2dB), connected to a Sony TCD-D10 DAT tape recorder (sampling frequency 48 kHz, flat response within the range 20–20000 Hz).

During playbacks, two observers were hidden at a few meters from the loudspeaker. One of the observer was in charge of emitting sounds; the other was blind to condition. In the case of localization experiments (two-loudspeakers set up), there was one observer hidden close to each loudspeaker. The reaction of the bird was assessed by both observers and the distance at which the bird approached the loudspeaker was visually assessed and categorized as being more than 10 m or less than 10 m.

### Sound stimuli

We built synthetic control by using the Synthesizer of Avisoft SASLab Pro software [31]. Its principle is to first use the spectrograms of natural songs as models, then scan the frequency contour and amplitude envelope at regular time intervals, and finally save the corresponding values into a WAV file. Experimental signals were made from these synthetic copies by selectively modifying acoustic values in the time and frequency domains. The synthetic continuous pure tone was built using Syntana software [32]. The 50m-propagated songs were obtained by performing transmission experiments as described below.

### Propagation experiments

A synthetic copy of a representative song of the white-browed warbler was broadcast and re-recorded at 6 m high (typical song post height) over four distances (control signal: 1.5 m; propagated signals: 25, 50, and 100 m which represents the extreme diameter of a territory; propagation transect chosen to be well representative of the forest environment). We analyzed the re-recorded sounds by comparing the propagated signals with the control. To assess modifications of the main amplitude fluctuations, signals envelopes were digitally filtered (150 Hz high-pass) and averaged (*n* = 6) for each propagation distances. Correlations (r values) between control and propagated signals envelopes and their significance were calculated using Bravais-Pearson product-moment. To assess modifications of the frequency modulation, signals spectrograms were averaged (*n* = 6) for each propagation distance, and compared using the digital spectrographic cross-correlation method [33], [34].

### Playback experiments 1: species-specific information

Experiments were performed between 06h15 and 10h30 (series of five consecutive experimental songs played at natural song rate), situated in the approximate center of the bird territory, at 2 meters high). Only one treatment per tested individual was done to avoid habituation (a total of 56 birds was tested). To limit pseudo-replication, synthetic copies of three songs from different individuals were used as original control songs. Modified experimental signals were made from these copies by digital synthesis using Avisoft SasLab Pro [31] and Syntana software [32]. Each of the control copies and their corresponding experimental signals were used in a balanced manner between the tested birds (i.e., one third of the birds were tested with signals built from one of the three original songs, another third of the birds were tested with signals built from another of the three original songs, the remaining birds were tested with signals built from the third original song). The behavioral responses observed during the playback trial and the three minutes following it, were compared with the bird behavior during the three minutes before the playback. Four response measures were scored:

- latency of response –in terms of approach, song and/or calls emitted in reply- (score 0: no response within 3 minutes after the end of the playback session; score 1: response between 1 and 3 minutes after the playback session; score 2: response during playback or within 1 minute after the playback session);
- approach to the speaker (score 0: none; score 1: >10 m; score 2: <10 m);
- variation of call emission rate (score 0: no variation; score 1: weak variation, i.e. up to 3 times the initial calling rate in terms of number of calls/minute; score 2: strong variation, i.e. more than 3 times the initial calling rate);
- variation of song emission rate (score 0: no variation; score 1: weak variation, i.e. until 3 times the initial singing rate in terms of number of songs/minute; score 2: strong variation, i.e. more than 3 times the initial singing rate).

Responses to control song and experimental signals were compared using Kruskal-Wallis tests followed by post-hoc non parametric multiple comparisons [35].

### Playback experiments 2: individual information

All the birds tested here were different from the ones used in the previous experiments. Each experiment consisted of two consecutive trials (paired test, one experiment per tested bird, n = 20), one presenting an original neighbor's song (control) and one presenting either an unfamiliar song or a modified neighbor's song (experimental). Each trial was composed of a series of five consecutive songs played at natural song rate; the loudspeaker being situated at the border between the tested bird territory and its neighbor's one, at 2 meters high. The order of trials presentation was balanced between the tests (time interval: at least 10 minutes). Behavioral responses to playback were assessed independently for each trial using the following behavioral scale: 1: the tested bird answered vocally to playback signals and approached at less than 10 meters from the loudspeaker, 2: the tested bird answered vocally but did not approach, 3: the tested bird showed no reaction to playback. A sign test was used to compare responses between neighbor's song and unfamiliar or modified song. If contiguous neighbors were tested with this protocol, tests were performed on different days and using different stimuli to avoid habituation.

### Playback experiments 3: ranging

All the birds tested here were different from those in the other experiment and each individual was challenged only once (n = 18). Experimental songs were recorded from remote individuals (not neighbors). Behavioral response to the experimental signal was assessed in term of latency of departure from the first loudspeaker (‘immediate departure’, i.e., departure during the emission of the experimental signal, or ‘delayed departure’, i.e., after the end of the experimental signal playback), and in term of approaching distance to the second loudspeaker (‘close approach’, i.e., at less than 10 m, or ‘distant approach’, i.e. more than 10 m). Fisher's exact test was used for comparisons.

### Playback experiments 4: azimuth assessment

The procedure was identical to that of experiment 3, except that the experimental signal emitted by the second loudspeaker was either a control song, either a song without AM (n = 18 birds tested, different than in previous experiments). Besides latency of departure and distance of approach, behavioral response was also assessed in term of directionality (‘correct direction’: the birds flew directly, with a precision of ± 20°, towards the second loudspeaker in reaction to the experimental signal; ‘incorrect direction’: the birds flew in a direction different of more than 20° to the second loudspeaker). Directionality was assessed by the observer situated close to the first loudspeaker. Fisher's exact test was used for comparisons.

All statistical tests have been performed using STATISTICA 6.1 software.

## Supporting Information

Audio S1 Territorial (control) song (211 KB wav file).
(0.22 MB WAV)

Audio S2 100 m propagated song (431 KB wav file).
(0.44 MB WAV)

Audio S3 Synthetic continuous pure tone (233 KB wav file)
(0.24 MB WAV)

Audio S4 FM slope multiplied by 1.5 (499 KB wav file).
(0.51 MB WAV)

Audio S5 FM slope divided by 2 (502 KB wav file).
(0.51 MB WAV)

Audio S6 Second half of the song (202 KB wav file).
(0.21 MB WAV)

Audio S7 Neighbor's control followed by time-shifted neighbor's song (2 MB wav file)
(2.05 MB WAV)

Audio S8 Neighbor's control followed by frequency-shifted neighbor's song (2 MB wav file)
(2.03 MB WAV)

Audio S9 Song without AM (511 KB wav file).
(0.52 MB WAV)
